# Supplementary material for: UV-Activated NO2 Gas Sensing: Photoactivated Processes on the Surface of Metal Oxides
Source: Nanomaterials (Basel). 2025 Nov 28;15(23):1795. doi: 10.3390/nano15231795 (PMC12693671; doi:10.3390/nano15231795)
Supplement: Supplementary file 1 [file nanomaterials-15-01795-s001.zip › nanomaterials-3966244-supplementary.pdf]

UV-Activated NO_2 Gas Sensing: Photoactivated Processes on the Surface of Metal Oxides

Pavel Kutukov [1,*], Daria Kurtina [1,2], Sergey Maksimov [1] and Marina Rumyantseva [1,*]

[1] Chemistry Department, Moscow State University, 119991 Moscow, Russia
[2] Federal Research Center Kazan Scientific Center RAS, 420111 Kazan, Russia
* Correspondence: kutukovps@my.msu.ru (P.K.); roum@inorg.chem.msu.ru (M.R.)

Supplementary materials

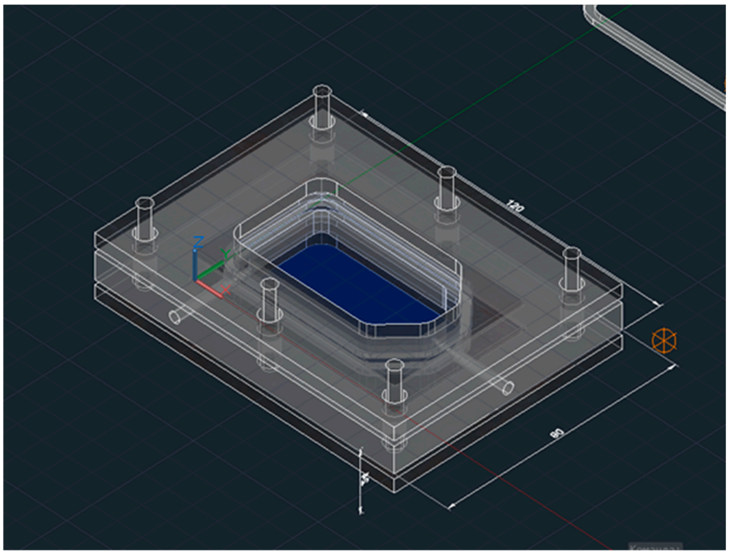

Figure S1. Flow-cell construction for mass-spectormetry investigations.

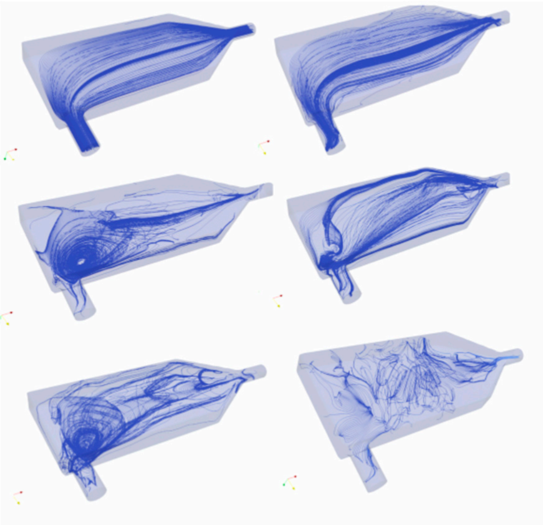

Figure S2. Flow simulation results, showing no extra-volume effects (no eddies in the corners) and instability of the laminar flow regime.

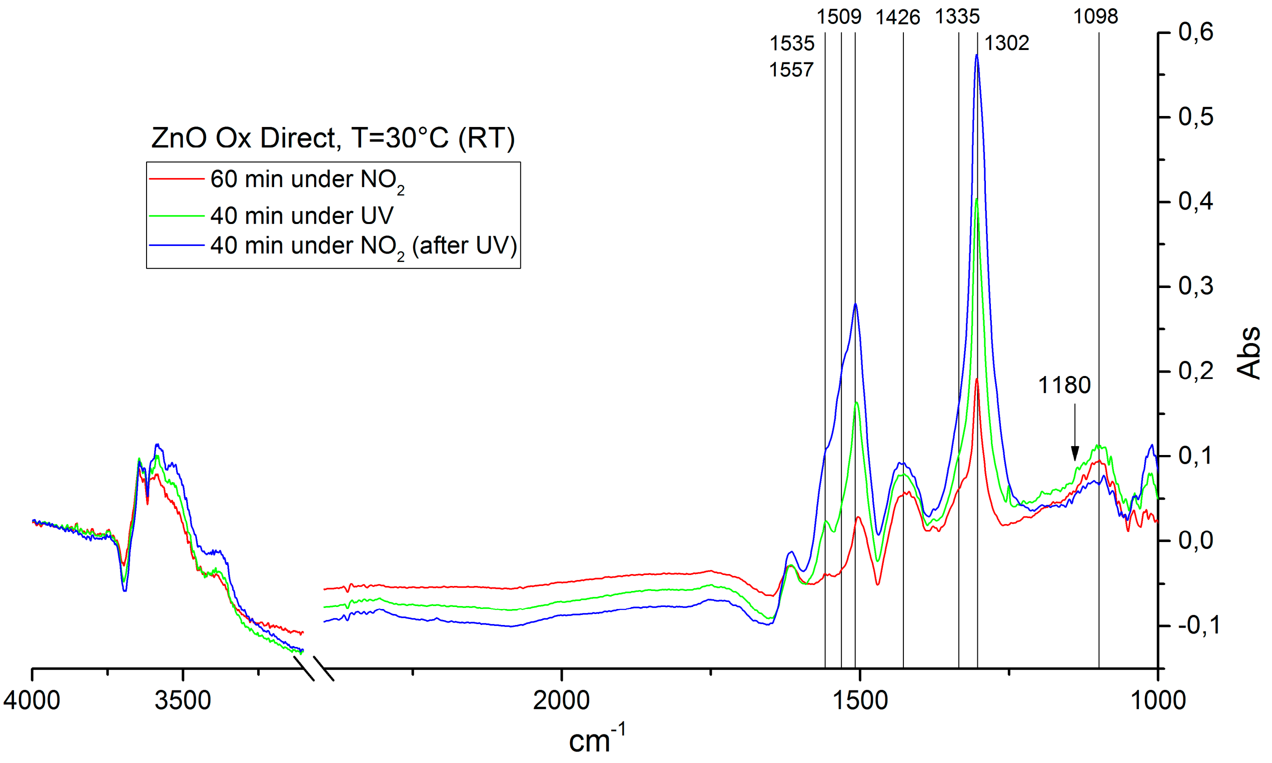

Figure S3. Chronological TR-DRIFT spectra for ZnO exposed to 100 ppm NO_2 at room temperature (with photoactivation).

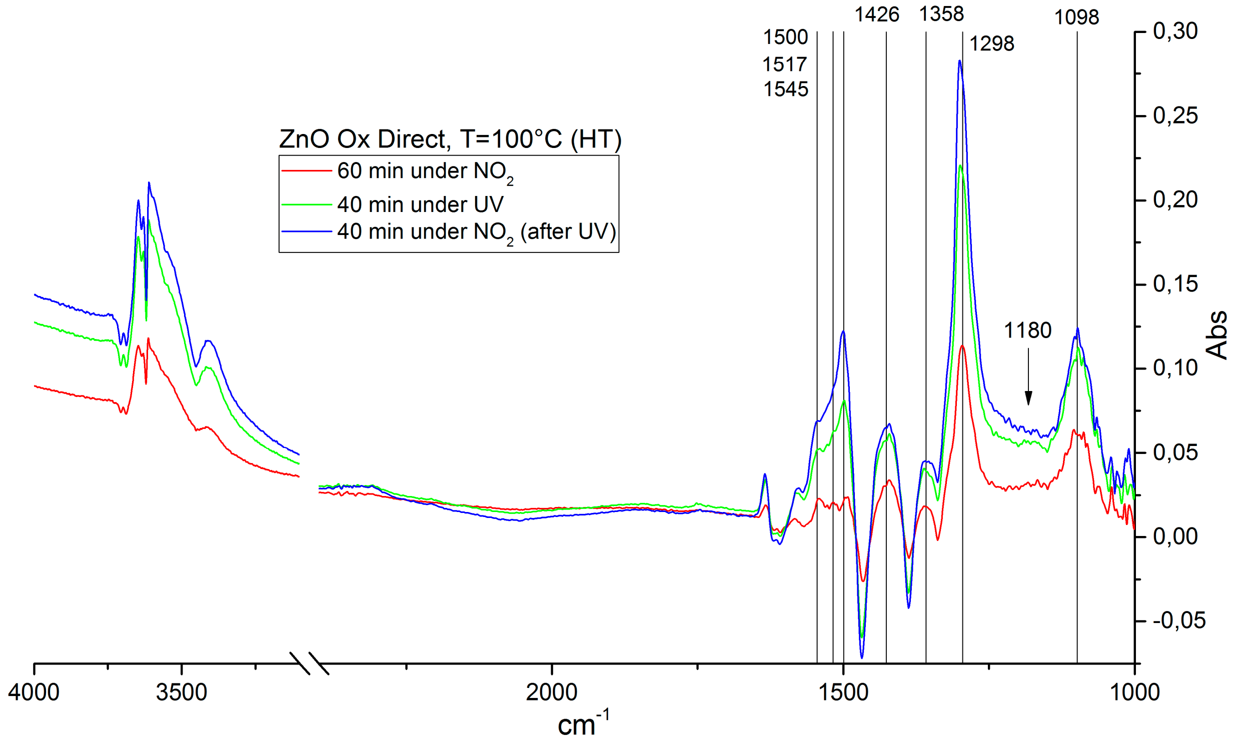

Figure S4. Chronological TR-DRIFT spectra for ZnO exposed to 100 ppm NO_2 at 100°C (with photoactivation).

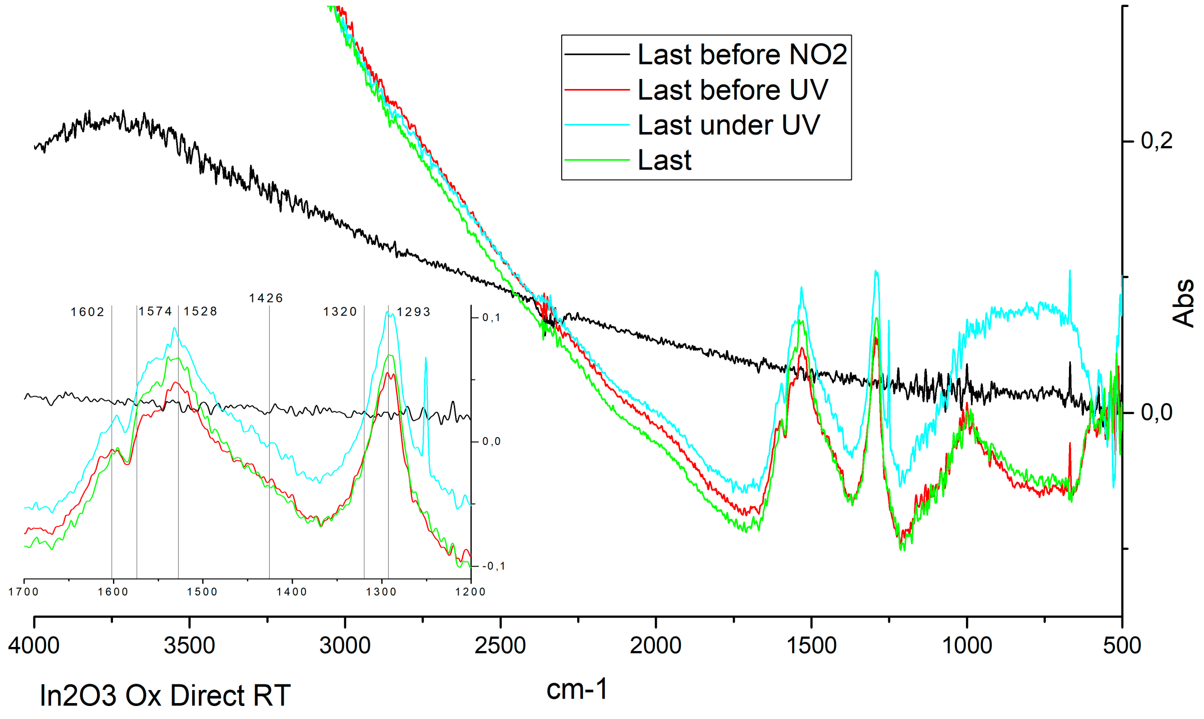

Figure S5. Chronological TR-DRIFT spectra for In_2O_3 exposed to 100 ppm NO_2 at room temperature (with photoactivation).

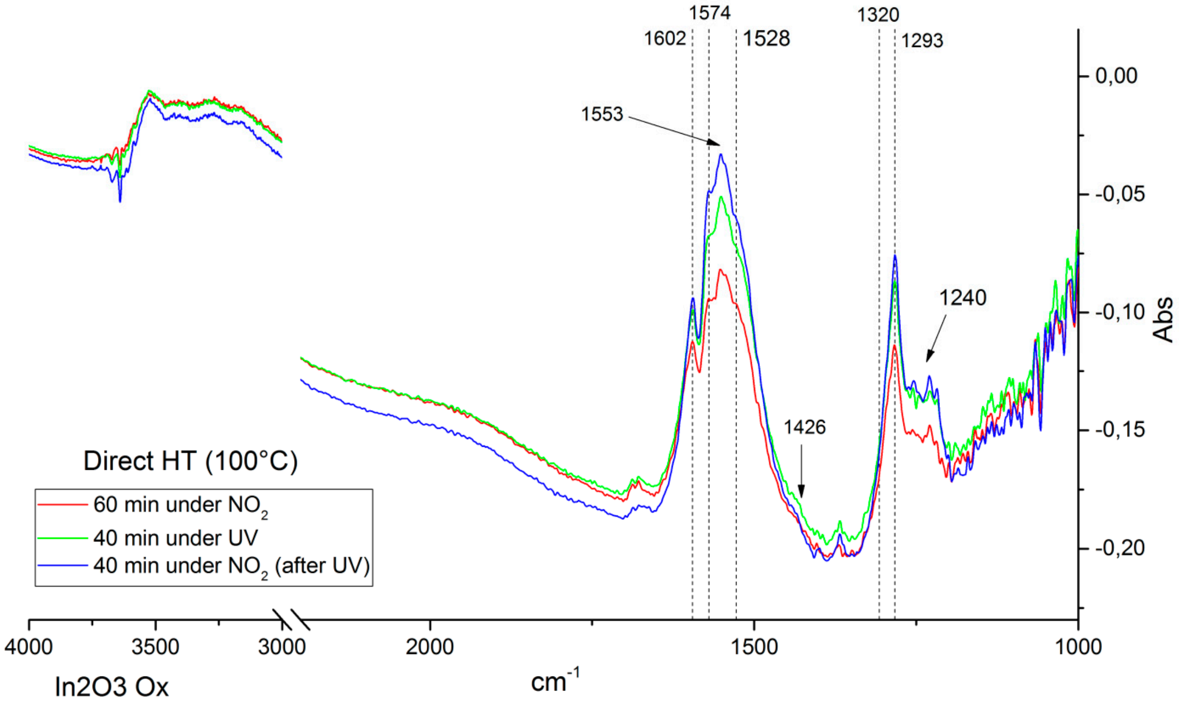

Figure S6. Chronological TR-DRIFT spectra for In_2O_3 exposed to 100 ppm NO_2 at 100°C (with photoactivation).

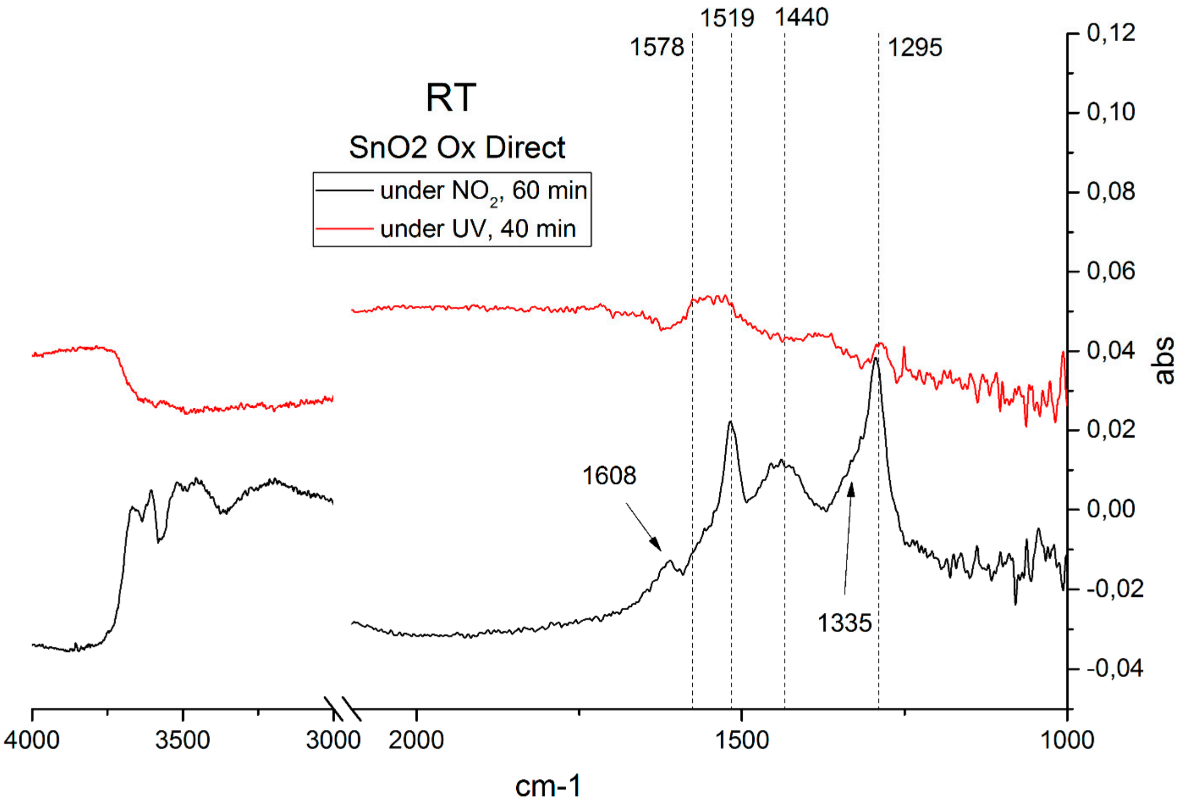

Figure S7. Differential TR-DRIFT spectra for SnO_2 exposed to 100 ppm NO_2 at room temperature (with photoactivation).

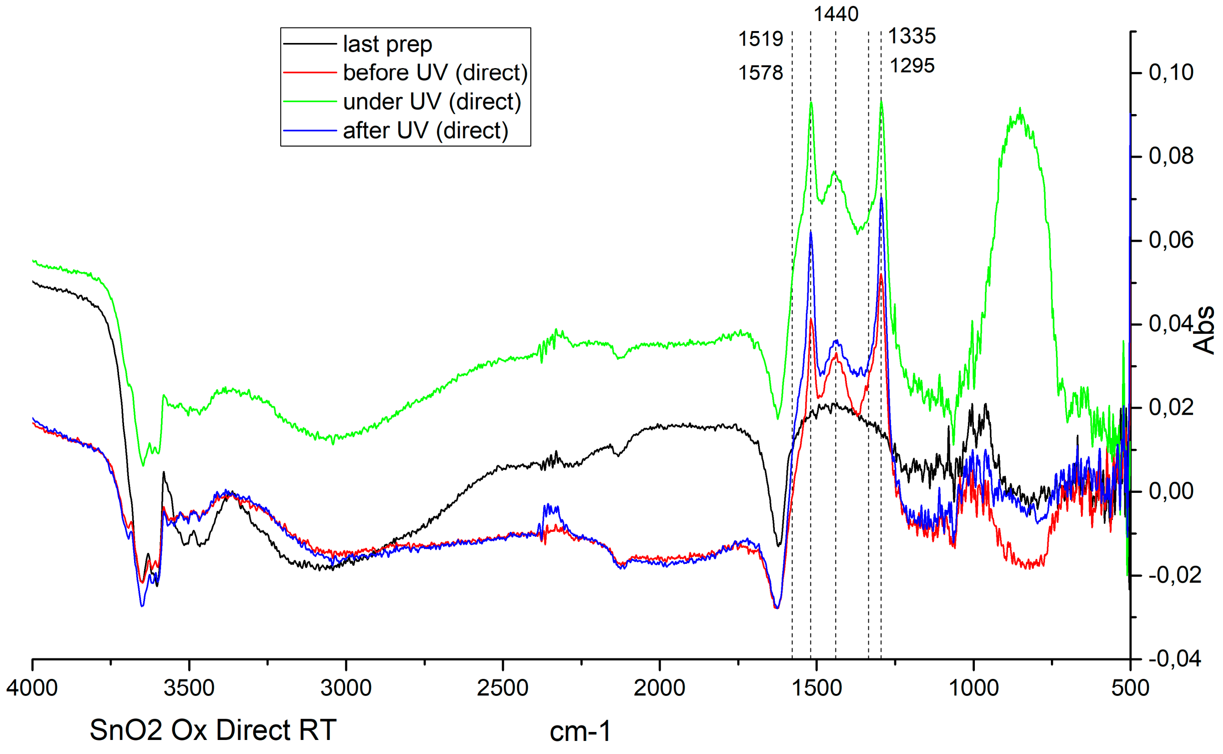

Figure S8. Chronological TR-DRIFT spectra for SnO_2 exposed to 100 ppm NO_2 at room temperature (with photoactivation).

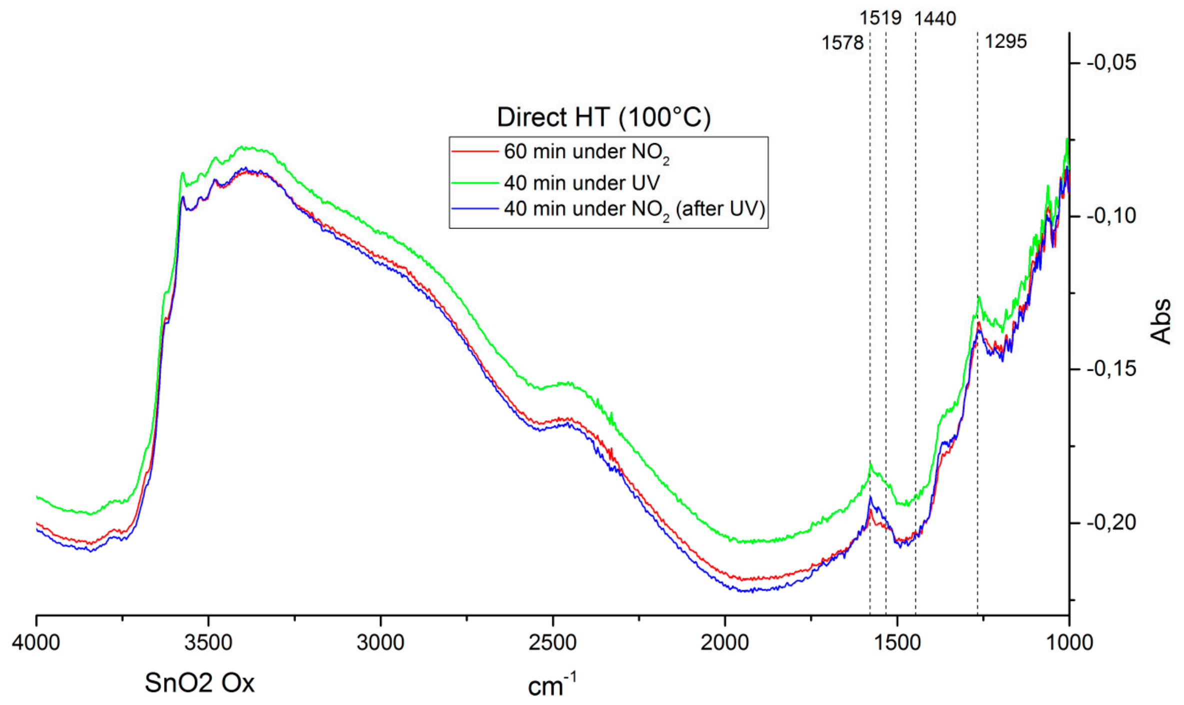

Figure S9. Chronological TR-DRIFT spectra for SnO_2 exposed to 100 ppm NO_2 at 100°C (with photoactivation).

Table S1. Calculated exponential time constants (in seconds) of sensor response and relaxation. Blank means only a single time constant is present (either t1 or t2). “NA” means no data is available. All values were calculated for 2 ppm NO_2 step. A 3 minute transient period was excluded from calculations.

Gas (response)				Air (relaxation)			
**t1**	**T, °C**			**t1**	**T, °C**		
**Dark**	**30**	**50**	**100**	**Dark**	**30**	**50**	**100**
ZnO	NA	200	91	ZnO	NA	147	110
In2O3	74	136	89	In2O3	120	105	78
SnO2	NA	NA	100	SnO2	NA	NA	74
**UV**	**30**	**50**	**100**	**UV**	**30**	**50**	**100**
ZnO	80	62	60	ZnO	92	86	66
In2O3	172	286	-	In2O3	54	52	38
SnO2	221	192	-	SnO2	68	64	38
Gas (response)				Air (relaxation)			
**t2**	**T, °C**			**t2**	**T, °C**		
**Dark**	**30**	**50**	**100**	**Dark**	**30**	**50**	**100**
ZnO	NA	4000	914	ZnO	NA	1246	809
In2O3	487	1043	509	In2O3	-	-	-
SnO2	NA	NA	602	SnO2	NA	NA	433
**UV**	**30**	**50**	**100**	**UV**	**30**	**50**	**100**
ZnO	541	570	701	ZnO	762	784	-
In2O3	636	2473	2400	In2O3	306	275	166
SnO2	1652	4536	1500	SnO2	311	242	212
